# Disease X ver1.0: COVID-19

**DOI:** 10.1071/MA20028

**Published:** 2020-05-27

**Authors:** Paul R Young

**Affiliations:** School of Chemistry and Molecular Biosciences, The University of Queensland, Qld, Australia. Email: p.young@uq.edu.au

## Abstract

The SARS-Cov2 has presented the world with a novel pandemic challenge requiring a rapid response. This article provides a May 2020 snapshot from Professor Paul Young, who is part of a group working with urgency on Australia’s leading COVID-19 candidate vaccine.

We first noted tweets about a new respiratory infection in Wuhan, China in late December 2019. At that time, we were about a year into a three-year grant funded by the Coalition for Epidemic Preparedness Innovations (CEPI). The goal of that grant was to establish a streamlined, Australia-based, rapid response vaccine pipeline to address the threat of emerging viral pathogens. The project was built around a patented platform technology that we had been developing here at the University of Queensland (UQ) for nearly 10 years^1^. We had already generated candidate subunit vaccines for 10 different viruses from a wide range of viral families and so were well placed to apply all that accumulated knowledge to this newly emerging virus. Initially, we viewed the task as an exercise to test the platform, not expecting the global spread that would follow. In those early days of January, we eagerly awaited the release of any viral sequence information. On 10 January 2020 the first full genome sequence of this new virus, a coronavirus like its predecessors SARS and MERS, was made public and overnight we had designed our first constructs.

We named our patented platform technology the Molecular Clamp. It was the brainchild of Keith Chappell, a post-doctoral scientist who had originally completed his PhD with me and then returned to my lab in 2011 after a post-doc stint in a leading respiratory syncytial virus (RSV) lab in Madrid. His task in Madrid, with the celebrated virologist José Melero, was to recombinantly engineer the RSV fusion protein F, to capture it in its pre-fusion form. The theory was that this form of the protein is what appears on the surface of the virus and so is the primary target of a protective antibody response. These proteins undergo a dramatic conformational change in driving the process of viral-host membrane fusion and in its post-fusion form, many of the epitopes recognised by antibodies on the native virion are hidden. Keith’s work in successfully producing a constrained pre-fusion form of F was instrumental in Melero’s team making the seminal observation that the majority of naturally acquired neutralising antibodies recognised the pre-fusion and not post-fusion form of F. This was a critical observation for vaccine design^2^. The problem was that his approach resulted in a protein that was not that stable.

When he returned to my lab it was to work in a relatively new area for us, virus-bacterial interactions, but he asked if he could also continue to work on the RSV F story. I had been involved with Biota for a number of years in the late 1990s, expressing RSV F as a target for antiviral drug design, and through that work we had discovered the second cleavage site for this protein. So, I was primed to be interested. Within that first year he came up with the idea of fusing the two heptad repeats of another fusion protein to the end of the target RSV fusion protein ectodomain. The highly stable six helical bundle that formed from their spontaneous folding and association provided a remarkably stable trimerisation domain. The irony is that it is the very stability of this post-fusion structural domain that we were able to re-purpose to stabilise the pre-fusion form of the protein. So began a long journey of unfunded research (consultancy revenue comes in handy), with Dan Watterson, another PhD graduate of my lab and returned post-doc, contributing substantially to what became the Molecular Clamp (MC). The three of us are co-inventors on the MC patent^1^. Despite numerous funding applications over subsequent years, including industry pitches, our first successful grant, specifically for this work was an NHMRC Project, submitted in 2017. Perseverance, or perhaps stubbornness is highly underrated, as so often is the basic science that underpins translational outcomes.

Also, in early 2017 I took a punt and booked a flight to Paris to attend the opening of a new organisation, CEPI, that I had only just heard about. It was a transformative experience for me. I have been passionate about contributing to neglected disease research all my working life, and had been involved in wonderfully collaborative and transformative research projects. But I had never felt as much positive energy as I felt at that meeting, full of leading academic researchers, innovative NGOs and small biotechs alongside large pharma, all committed to finally answering the World Health Organization (WHO) call to deliver on a global preparedness strategy to deal with emerging pathogen threats. CEPI’s mission was articulated at that meeting; to stimulate and accelerate the development of vaccines against emerging infectious diseases and enable equitable access to these vaccines for people during outbreaks. In addition to specific virus targets they also support platform technologies that could be applied to newly emerging pathogens, referred to by the WHO as Disease X.

On my return, Keith and I committed to an application to their first call for vaccine strategies targeting selected pathogens from the WHO Blueprint Priority disease list. This first application was not successful. However, CEPI liked what they saw in our proposal and asked us to submit to the next call, which was to support platform technologies that could be applied to multiple pathogen targets. The call had a number of key criteria that needed to be met, the most notable being a 16-week timeline from pathogen discovery to delivery of sufficient vaccine to enter a Phase 1 clinical trial. A challenging ask, but one we felt we could meet, given the seven years of development we had already put into our MC approach. Our application brought together colleagues from the ANU, the Doherty Institute, University of Hong Kong and CSIRO teams at both the protein manufacturing facility at Clayton in Melbourne and the AAHL facility in Geelong. To prove the technology, we needed to generate three separate vaccines, two for ‘demonstrator’ targets, i.e. ones for which existing vaccines or technology was already available to compare, and one emerging pathogen. We chose influenza and RSV for our first two targets and, fortuitously as it would turn out, the coronavirus MERS for the emerging pathogen. We also suggested in our grant proposal that in our last year of the three year grant we should be subjected to a stress test. We would be supplied with an unknown viral sequence, from which we needed to design, develop, test and manufacture enough vaccine to enter a Phase 1 clinical trial.

That was meant to happen in 2021, but we received that first, very real ‘stress test’ sequence on 10 January this year. The first constructs were designed within the first 24 hours. On 21 January we received a formal request from CEPI to begin full development and manufacture of a vaccine candidate. Within three weeks of receiving the initial SARS-CoV-2 sequence we had chosen a lead construct. We went on to design, express and test more than 200 different constructs by the end of the 4th week, but we ended up moving forward with that first excellent lead candidate. A model of the clamped, trimeric pre-fusion SARS-CoV-2 Spike protein that we have generated as our vaccine candidate is shown in Figure 1*a*. Figure 1*b* shows the UQ leadership team for the CEPI project.

**Figure 1.**
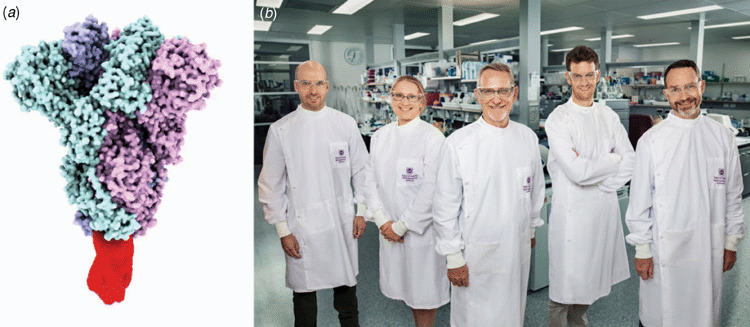
(*a*) Structural model of the trimeric SARS-CoV-2 Spike protein ectodomain (prepared by D Watterson), stabilised by the Molecular Clamp (red). (*b*) The UQ CEPI leadership team (L to R): Dan Watterson, lead researcher; Christina Henderson, Project Manager; Paul Young, Project Co-Lead; Keith Chappell, Project Co-Lead; and Trent Munro, Project Director.

The months that followed this early work have essentially been 24/7 for the whole team of about 20 UQ researchers, as well as all our colleagues in our partner institutions. It has been a revelation. Despite the immense workload, everyone has remained engaged and positive, and Zoom has become our constant companion. There have certainly been challenges along the way, but we have managed to keep to our original timeline of a start date for the Phase 1 clinical trial in early July 2020. Unlike some of the other candidate vaccines being developed globally, we have elected to complete all of our pre-clinical safety and efficacy studies prior to entering human clinical trials. At the time of writing, we have completed early mouse immunogenicity studies, which showed that the vaccine was able to induce highly potent neutralising antibody responses against live SARS-CoV-2, performed in collaboration with our colleagues at the Doherty. While mice are obviously not humans, the levels of neutralising antibody induced was substantially higher than that seen in recovered COVID-19 patients and so we are hopeful that we may be able to induce even higher levels of antibody with our vaccine than that induced by natural infection – it is early days, but the data are promising. We have been substantially assisted by large pharma (GSK, CSL and Dynavax) reaching out to us to offer their tried and tested adjuvants for this work. We have also now entered our vaccine into toxicology and animal protection studies, both of which should reach data points by June that will allow us to enter our Phase 1 study on schedule.

With the global race on, and more than 100 vaccines in development, we have also encountered challenges such as limited Australia-based capacity to support critical, high-level containment, animal challenge studies. CSIRO’s AAHL facility had moved quickly to begin ferret protection studies on vaccine candidates from two international groups (Oxford University and Inovio) and so was not available for our work. However, we were able to reach out to Viroclinics Xplore in The Netherlands and at the same time, expand the number of species we could test, as well as the overall scope of the studies.

Like everyone else, we have had to adjust to COVID-19 reaching our shores. By mid-March, the university was starting to shut down as many began working from home and practicing physical distancing (I still prefer that terminology to social distancing). We obviously needed to remain at work and in the lab and so, on 20 March we met for the last time as a single group, with appropriate distancing, and split into two teams that would no longer physically interact. That way, if one person fell ill we would not lose the whole group to home isolation. It has been a strange time at the university, to be in the middle of semester with all teaching now online and virtually no one on campus.

The timeline for development of a vaccine for COVID-19 has been a topic of much debate. The typical timeline for vaccine development, from conception to licensure is anything from 10–20 years, with five years being the most impressive to date. Regardless, most commentators have been suggesting a 12–18 month timeline. This is a challenging ask, as there can be no corner cutting when it comes to safety and efficacy. Accelerated timelines and adaptive design for clinical trials, expedited regulatory approval, accelerated manufacturing and early emergency and compassionate use are all part of the strategies for the early delivery of viable vaccines. In early March we developed a strategy to uncouple manufacturing from the typical pipeline of vaccine development, with the intention to run full-scale manufacturing alongside the clinical trials and not wait for confirmation of efficacy. It is a financially risky strategy, but one that could deliver significant vaccine doses, initially for emergency use and then immediately once regulatory approval was received. This would require a significant early funding boost and so we submitted a proposal that outlined this parallel development plan (Figure 2). The proposal was jointly funded by the Queensland government, the Federal government, and generous philanthropic support from Foundations and the community. The overall level of support and positive feedback and encouragement we are receiving has been extraordinary. Everything from the major Foundation donations, a number of whom have not funded in the medical space previously, to the letters of support we have received from school children and the smaller, but no less important donations, such as the $6.50 that was sent to us by one child in Victoria from his ‘share’ jar, have all been truly inspirational.

**Figure 2.**
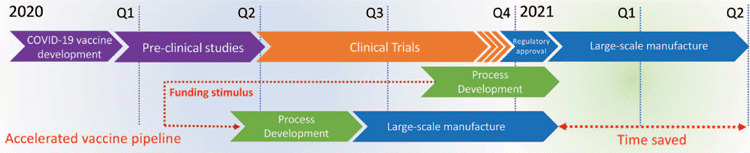
Schematic of the UQ COVID-19 subunit vaccine development pipeline. Funding stimulus has allowed us to advance and accelerate vaccine manufacture, cutting some 6 months off the expected vaccine delivery timeline.

The development of our vaccine is now the primary focus of the team and is continuing at pace, with all members of our consortium managing a range of variables that we continually need to adjust. What would normally take years to develop and finesse, we have only weeks and months to progress. But groups all over the world, developing the more than 100 candidate vaccines that are currently in play, will be having similar issues. The major triage point is coming soon: the shift to large-scale manufacture. There is limited global capacity available and it is likely that only 3–4 vaccines will make their way through this transition point. We are hopeful that ours will be one of those vaccines that makes it through the months ahead, with its use ultimately contributing to the control of this once-in-a-lifetime pandemic.

## Conflicts of interest

The author declares no conflicts of interest.
